# Mucosal-Associated Microbiota Other Than Luminal Microbiota Has a Close Relationship With Diarrhea-Predominant Irritable Bowel Syndrome

**DOI:** 10.3389/fcimb.2020.515614

**Published:** 2020-11-02

**Authors:** Min Yang, Gaichao Hong, Yu Jin, Ying Li, Gangping Li, Xiaohua Hou

**Affiliations:** Division of Gastroenterology, Union Hospital, Tongji Medical College, Huazhong University of Science and Technology, Wuhan, China

**Keywords:** irritable bowel syndrome with diarrhea, mucosal-associated microbiota, luminal microbiota, clinical symptoms, structural and metabolic alterations

## Abstract

Studies have linked dysbiosis of gut microbiota to irritable bowel syndrome (IBS). However, dysbiosis only referring to structural changes without functional alteration or focusing on luminal microbiota are incomplete. To fully investigate the relationship between gut microbiota and clinical symptoms of Irritable Bowel Syndrome with Diarrhea (IBS-D), fecal samples, and rectal mucosal biopsies were collected from 69 IBS-D patients and 20 healthy controls (HCs) before and during endoscopy without bowel preparation. 16S rRNA genes were amplified and sequenced, and QIIME pipeline was used to process the composition of microbial communities. PICRUSt was used to predict and categorize microbial function. The composition of mucosa-associated microbiota (MAM) was significantly different in IBS-D patients compared to HCs; while no difference in luminal microbiota (LM). MAM, but not LM, was significantly positively correlated with abdominal pain and bloating. A greater number of MAM functional genes changed in IBS-D patients than that of LM compared with HCs. Metabolic alteration in MAM not in LM was related to abdominal pain and bloating. There was a close relationship between the composition and function of MAM and clinical symptoms in IBS-D patients which suggests the important role of MAM in pathogenesis and therapies in IBS-D and it should be highlighted in the future.

## Introduction

Irritable bowel syndrome (IBS) is a common gastrointestinal disorder with a high incidence, persistent symptoms, and limited therapeutic options, and greatly affects the quality of life (Ford et al., 2017). Dysbiosis of gut microbiota has been indicated in IBS in recent years (Rajilić-Stojanović et al., 2015). However, while studies have reported significant alterations in the gut microbiota of IBS patients, the relationship between gut microbiota and clinical symptoms of IBS is unclear.

Studies that have focused on the relationship between luminal microbiota (LM) and IBS have shown inconsistent results (Rajilic-Stojanovic et al., 2011; Carroll et al., 2012b; Jalanka-Tuovinen et al., 2014; Tap et al., 2017). Additionally, these studies have used a small number of samples and the selection criteria and detection methods have introduced bias (Duan et al., 2019). Recent findings on the relationship between mucosal-associated (MAM) and IBS have not reported significant changes in MAM, likely since mucosal specimens are difficult to obtain which limits the number of samples (Carroll et al., 2012a; Rangel et al., 2015; Maharshak et al., 2018). Also, the mixed subtypes of IBS and disruption of MAM caused by bowel preparation can affect results (Rangel et al., 2015).

While most researchers have evaluated changes in MAM and LM structure, few have focused on microbiota function. Intestinal MAM and LM are two diverse ecosystems with different structures and functions (Van den Abbeele et al., 2011). MAM plays an important in health and disease. Given that MAM exists in mucus, it can mediate the integrity of mucus layer which acts as a first line of defense barrier to pathogenic microbes (Desai et al., 2016). It also affects gut immune function through intestinal epithelial cells and immune cells (Wang et al., 2016), while LM plays a role in digestion and absorption of carbohydrates (Borgo et al., 2018). These two types of gut microbiota play different roles in different diseases.

Currently, no study exists on the relationship between microbiota function and clinical symptoms in IBS. Since the role of gut microbiota in disease is dependent upon microbiota function, it is necessary to investigate the relationship between the structure and function of gut microbiota.

In this study, we evaluated Irritable Bowel Syndrome with Diarrhea (IBS-D), a subtype of IBS, to avoid confounding effects from other subtypes. We also harvested samples without bowel preparation to avoid its impact on MAM. We obtained as many fecal and mucosal samples as possible and applied next-generation sequencing to analyze MAM and LM. We also evaluated the relationship between clinical data and the structure and function of flora (including MAM and LM) to clarify whether MAM or LM changed significantly and which bacteria may be involved in the clinical symptoms through which functional genes.

## Materials and Methods

### Participant Recruitment

The Institutional Ethical Review Committee of Huazhong University of Science and Technology approved this study (H20080304), and all participants signed informed consent before sample collection. Experienced gastroenterologists recruited patients aged between 20 and 60 years in an outpatient room according to the inclusive and exclusive criteria. Patients had normal colonoscopy report within 2 years. Sixty-nine IBS-D patients (mean age: 36.61 ± 10.92 years) who fulfilled the Rome III criteria were recruited from the Division of Gastroenterology, Union Hospital, Tongji Medical College, Huazhong University of Science and Technology in Wuhan, China (Table 1). The Rome III criteria include recurrent abdominal pain or discomfort at least 3 days per month in the last 3 months with symptom onset at least 6 months before diagnosis associated with 2 or more of the following: improvement with defecation; onset associated with a change in frequency of stool; onset associated with a change in form of stool. IBS-D was defined by loose (mushy) or watery stools ≥25% (Longstreth et al., 2006). Exclusion criteria included having upper gastrointestinal symptoms (such as heartburn, acid reflux, epigastric fullness, and distention), taking of probiotics, antibiotics, or Proton-Pump Inhibitors (PPI) within the previous 3 months or diagnosis with other severe diseases. Clinical data such as age, sex, BMI, pain, bloating, symptom frequency, symptom location (9 abdomen regions), duration, stool frequency, stool form, defecation urgency, and total score of symptoms were collected by using a completed form which based on gastrointestinal symptom rating scale-IBS(GSRS-IBS) (Li et al., 2018). The stool form was evaluated by the Bristol Stool Form (Li et al., 2018). Symptom severity was evaluated using the GSRS-IBS as we used in previous study (Li et al., 2018) (1 = no discomfort at all; 2 = minor discomfort; 3 = mild discomfort; 4 = moderate discomfort; 5 = moderately severe discomfort; 6 = severe discomfort; 7 = very severe discomfort). Twenty healthy volunteers (mean age 36.35 ± 10.41 years) without GI symptoms or history of an organic disease were recruited through advertisements (Table 1).

**Table 1:** Clinical characteristics of subjects.

	**HCs (*n* = 20)**	**IBS-D patients (*n* = 69)**
Age	36.35 ± 10.41	36.61 ± 10.92
Gender (M/F)	7/11	47/22
BMI	21.36 ± 2.32	21.61 ± 3.48
Total score of symptom	NA	4.39 ± 1.85
Symptom frequency (per/month)	NA	3.98 ± 0.98
Duration (years)	NA	7.44 ± 7.32
Pain rate (%)	NA	66.70%
Bloating rate (%)	NA	75.40%
Stool frequency (per/day)	1 (1–2)	3 (2–5)
Bristol stool form	4 (3–4)	6 (5–7)
Defecation urgency rate	NA	60%

### Sample Collection

In this study, we investigated the mucosal microbiota (rectal biopsy samples) and the paired luminal microbiota (fecal samples) in IBS-D patients and healthy controls (HCs). None of the participants underwent bowel preparation or used antibiotics or probiotic 3 months before sample collection. Rectal mucosa was collected with the aid of endoscopy (Olympus, Japan) as described previously (Li et al., 2018), and feces were collected on the same day before the endoscopy. Samples were stored at −80°C until further analysis. A total of 61 mucosal samples (15 HCs and 46 IBS-D patients) and 87 luminal samples (18 HCs and 69 IBS-D patients) were collected from 89 participants.

### DNA Extraction and 16S rRNA Gene Sequencing

Genomic DNA was extracted from feces and biopsies using the FastDNA SPIN Kit (Tiangen, Beijing, China) according to the manufacturer's instructions. The V1–V3 regions of the 16S rRNA gene were amplified using universal primers (Zhou et al., 2013) (the forward primer containing the sequence “AGAGTTTGATCCTGGCTCAG” and the reverse primer containing the sequence “TTACCGCGGCTGCTGGCAC”) and fusion primers (the forward primer containing the sequence “454adapter-mid-AGAGTTTGATCCTGGCTCAG” and the reverse primer containing the sequence “454adapter-TTACCGCGGCTGCTGGCAC”). PCR was carried out using 25 μl reactions with 2 μl of DNA template, 10 μM of each primer, 0.125 μl of Takara Pyrobest polymerase (Takara Biotechnology Co., Ltd, Japan), 2.50 μl of 10× buffer, 2.5 mM dNTPs, and 16.375 μl of distilled water. The PCR cycling parameters were as follows: 4 min initial denaturation at 94°C, 27 cycles of denaturation at 94°C for 30 s, annealing at 55°C for 45 s, extension at 72°C for 1 min, and a final extension at 72°C for 7 min. PCR products were separated by 1.5% agarose gel electrophoresis in 1× TAE and purified using the Qiagen QIAquick Gel Extraction Kit (Qiagen Gmbh, Germany). All amplicons were then sequenced using a 454/Roche GS FLX platform at Personal Biotechnology Co., Ltd. (Shanghai, China).

### Bioinformatics and Statistical Analysis

Raw sequences were loaded into the QIIME analysis software and carried out using the default pipeline (Caporaso et al., 2010); the lengths of the sequences were between 200 and 1,000 nt, with a mean window quality score above 25, a maximum homopolymer run less than six, no ambiguous bases, and no mismatches in the primer. Similar sequences were clustered at a 97% sequence identity into operational taxonomic units (OTUs), which could be treated as sequence-based bacterial divisions using the Ribosomal Database Project classifier. We used the QIIME pipeline (Caporaso et al., 2010) to analyze the α-diversity (Observed OTU number, Shannon, Chao, and Ace) and β-diversity (Bray-Curtis and UniFrac distances) (Lozupone and Knight, 2005) based on the OTUs and its taxonomy information. The distance matrix of all the samples based on PCoA (Principal Coordinate Analysis) was performed by Emperor on the QIIME platform (Vazquez-Baeza et al., 2013). PERMANOVA (Permutational multivariate analysis of variance) with Bray-Curtis distance was used to compare the microbial community structure. PICRUSt (Langille et al., 2013) was used to predict and categorize the gene function of mucosal and luminal microbiota. The microbial feature differences between the two groups were performed using linear discriminant analysis (LDA) effect size (LEfSe) method (http://huttenhower.sph.harvard.edu/lefse/); a significance alpha of 0.05 was used for all samples (Segata et al., 2011). Differential analysis was performed using the Mann-Whitney U test by SPSS version 19.0 for Windows (SPSS Inc., Chicago, IL). Spearman's correlation test and Mantel's tests were performed in R (version 3.3.1). Benjamini-Hochberg false-discovery rate (FDR) was used to adjust the *P*-value in the case of multiple testing and q values <0.1 were considered statistically significant. All sequence data in this study are available in the GSA database (accession number: PRJCA002555).

## Results

### Characteristics of Sequencing Data

A total of 1,595,641 raw sequences were obtained from 149 samples (biopsies: *n* = 61; feces: *n* = 87), and 1,535,326 high quality reads remained following quality trimming and chimera checking for downstream analysis, accounting for 96.22% of the valid reads with an average of 10,709 reads per sample. The median read length was 499 base pairs (range: 173–933). The average values of Good's coverage were 98.9% for all samples, indicating sufficient sequencing depth to investigate MAM and LM in IBS-D patients.

### Structure of MAM and LM in IBS-D Samples

**Figure 1:**
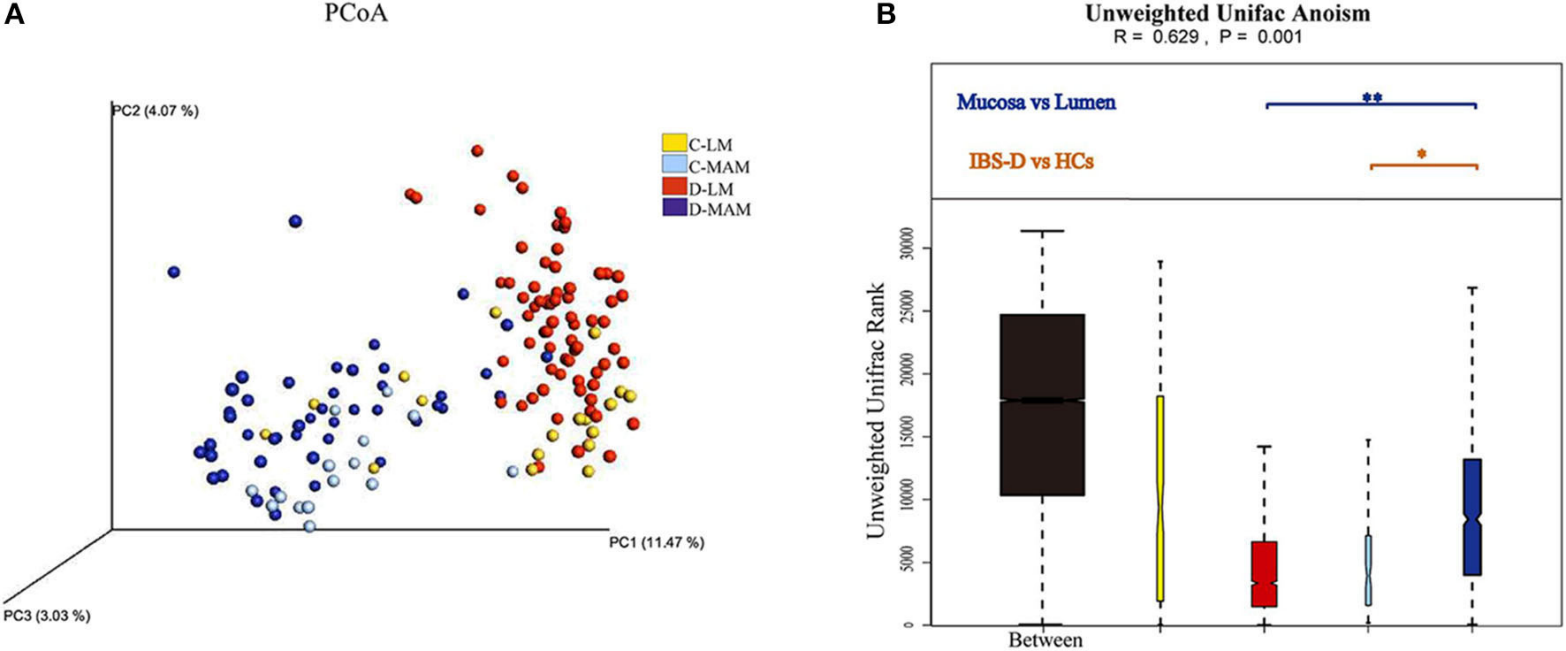
The global microbial structures of MAM and LM. The PCoA **(A)** based on unweighted Unifrac distance **(B)** showed a clear separation between MAM and LM in IBS-D patients. A similar separation also had been seen in MAM between IBS-D patients and HCs (C-LM, luminal microbiota in healthy controls; D-LM, luminal microbiota in IBS-D; C-MAM, mucosa-associated microbiota in healthy controls; D-MAM, mucosa-associated microbiota in IBS-D) (^*^P < 0.05, ^**^P < 0.01.

Analysis of the global microbial structures resulted in a clear separation of the PCoA based on unweighted Unifrac distance between MAM and LM both in IBS-D patients and HCs (Anosim R = 0.781, *P* = 0.001; R = 0.495, *P* = 0.001) (Figure 1). A similar separation was found in MAM between IBS-D patients and HCs (Anosim R = 0.36, *P* = 0.001); however, there was no difference in LM between IBS-D patients and HCs (Anosim R = −0.047, *P* = 0.785) (Figure 1). Bray-Curtis distance based on bacterial genus relative abundance was used to evaluate the difference between different groups. Permutational multivariate ANOVA (PERMANOVA) tests showed that the signatures of MAM in IBS-D patients and HCs were distinct (*P* = 0.039; PERMANOVA) while that no difference in LM between IBS-D patients and HCs (*P* = 0.38; PERMANOVA).

MAM had a higher diversity (Chao, Ace, and Shannon) than LM across all participants (Table 2). And LM had lower diversity (Shannon) in IBS-D patients compared to HCs, however, there was no difference in the diversity of MAM between IBS-D patients and HCs (Table 2).

**Table 2:** The microbial diversity of MAM and LM in all the subjects.

			**OTU**	**Chao**	**Ace**	**Shannon**
Mean ± SE		C-LM	666.44 ± 51.90	75.11 ± 11.15	76.92 ± 10.67	2.28 ± 0.10
		D-LM	715.16 ± 24.83	55.84 ± 1.72	59.27 ± 2.14	1.92 ± 0.06
		C-MAM	660.07 ± 39.3	154.81 ± 8.44	160.57 ± 8.57	2.74 ± 0.09
		D-MAM	762.5 ± 38	170.74 ± 8.7	174.19 ± 8.85	2.72 ± 0.1
*p*-value	HCs vs. IBS-D	LM	0.12	0.71	0.52	0.03
		MAM	0.07	0.3	0.35	0.7
	LM vs. MAM	Control	0.66	0	0	0.001
		IBS-D	0.4	0	0	0

*C-LM, luminal microbiota in healthy controls; D-LM, luminal microbiota in IBS-D; C-MAM, mucosa-associated microbiota in healthy controls; D-MAM, mucosa-associated microbiota in IBS-D*.

**Figure 2:**
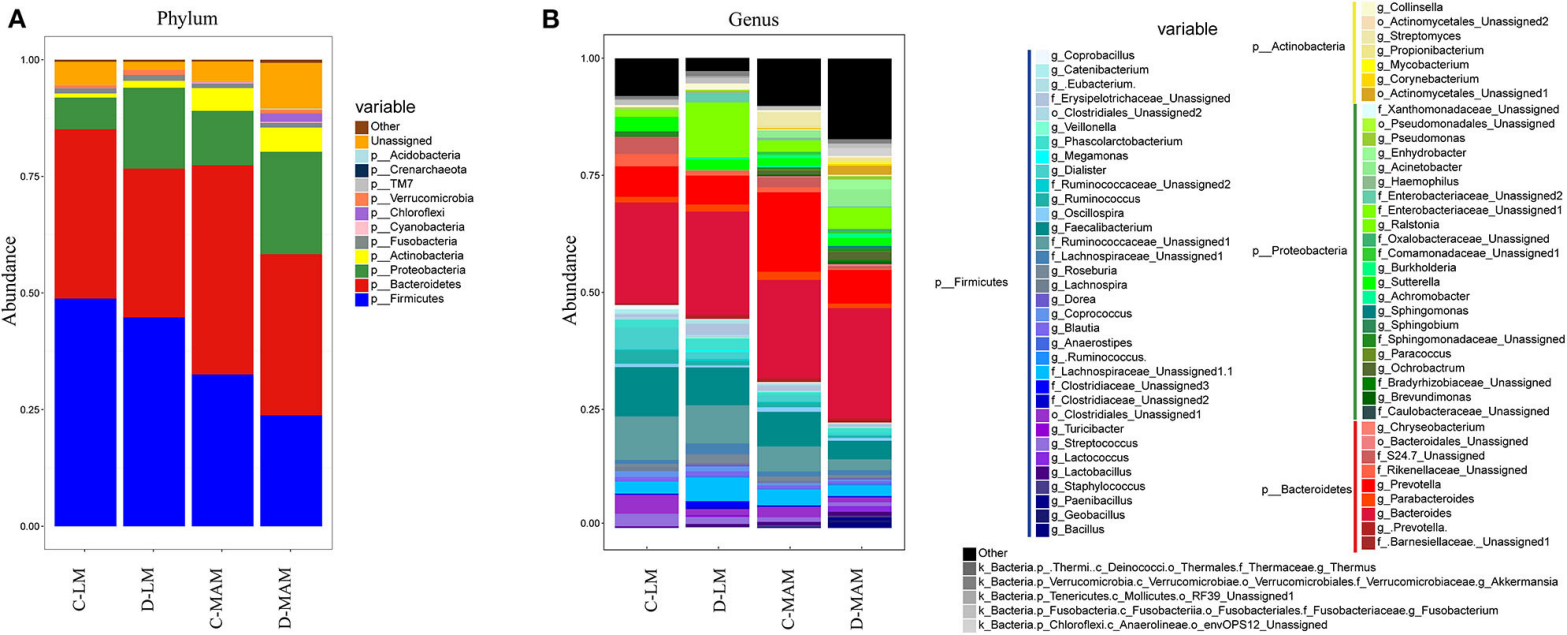
The microbial composition of MAM and LM. **(A)** The LM and MAM microbial composition at the phyla level and **(B)** at the genera level (C-LM, luminal microbiota in healthy controls; D-LM, luminal microbiota in IBS-D; C-MAM, mucosa-associated microbiota in healthy controls; D-MAM, mucosa-associated microbiota in IBS-D).

**Figure 3:**
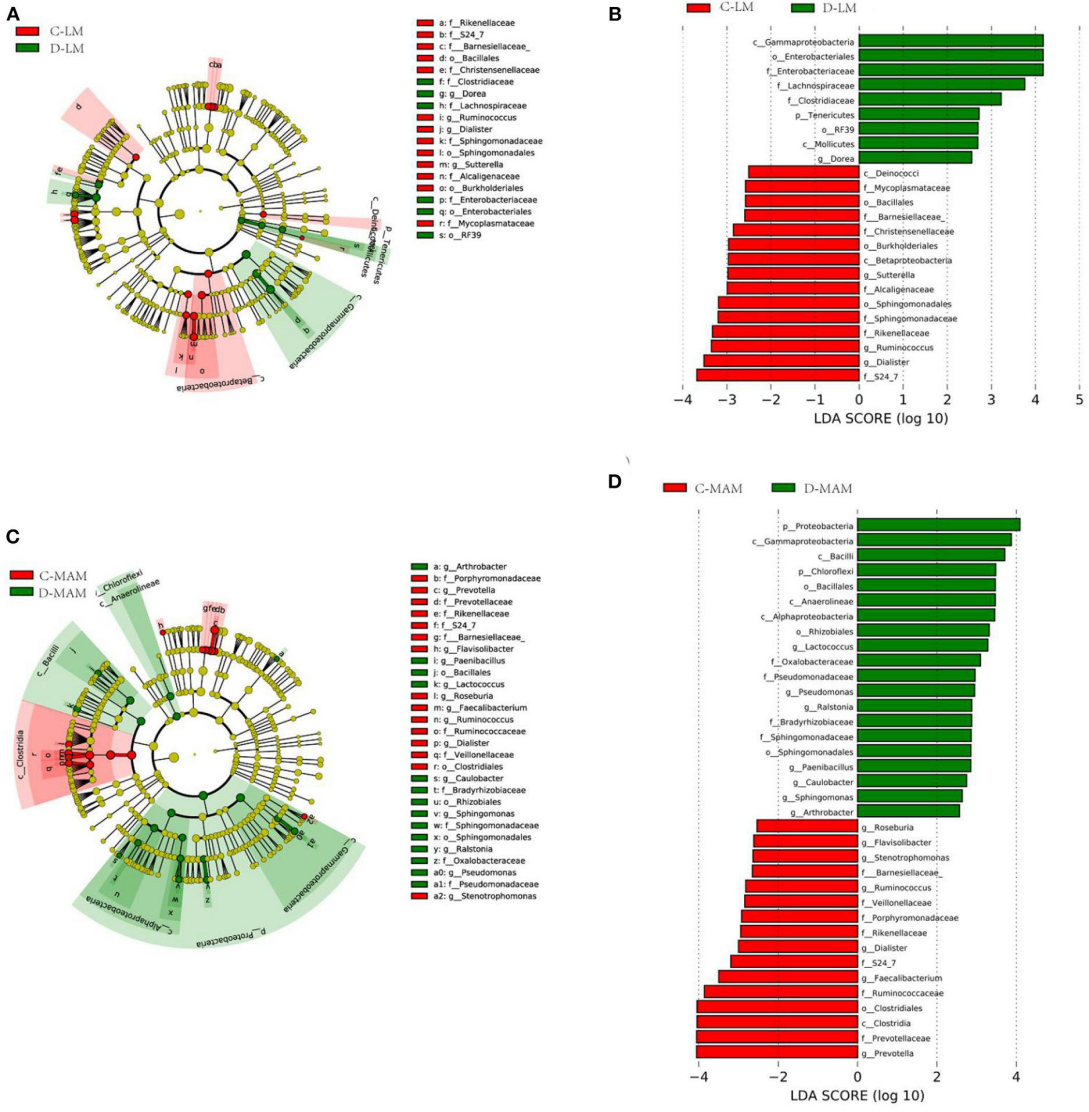
LefSe identified the taxons with differences between IBS-D patients and HCs. Taxonomic cladogram obtained from LEfSe analysis: **(A)** LM and **(C)** MAM. (Red) taxa enriched in HCs, (Green) taxa enriched in IBS-D patients. Only the LDA scores of taxa over 2.5 are shown **(B)** LM and **(D)** MAM (C-LM, luminal microbiota in healthy controls; D-LM, luminal microbiota in IBS-D; C-MAM, mucosa-associated microbiota in healthy controls; D-MAM, mucosa-associated microbiota in IBS-D).

Basing on the information classified by the RDP classifier and the OTUs taxonomy information, there were 41 phyla and 887 genera in the samples. Among them, 11 phyla were identified as predominant phyla (average abundance was over 0.1%) and 47 genera were predominant genera (average abundance was over 0.1%) (Figures 2, 3). At the phylum level, the MAM of IBS-D patients had a higher abundance of *Proteobacteria* (22.0 ± 2.4% vs. 11.6 ± 1.4%; *P* = 0.004) and *Chloroflexi* (1.8 ± 1.6% vs. 0.06 ± 0.02%; *P* = 0.04) and a lower abundance of *Firmicutes* (23.8 ± 1.7% vs. 32.6 ± 2.5%; *P* = 0.01) compared with HCs. The LM of IBS-D patients had increased *Tenericutes* (0.38 ± 0.38% vs. 0.2 ± 0.11%; *P* < 0.001) and decreased *Thermi* (0 ± 0% vs. 0.2 ± 0.18%; *P* < 0.001) compared to HCs. The IBS-D patients had 16 predominant genera in MAM and 7 predominant genera in LM, which differed significantly from HCs (Supplementary Table 1). The total abundance of significantly different predominant phyla in MAM was markedly higher than that in LM (46.8 vs. 3.9%; *P* < 0.001), similar to the tendency seen in the predominant genera above (MAM vs. LM: 21.2 vs. 8.1%; *P* < 0.001). MAM composition changed more than LM in IBS-D patients compared to HCs.

### Correlation Between the Structure of Microbiota and Clinical Manifestation in IBS-D Patients

**Figure 4:**
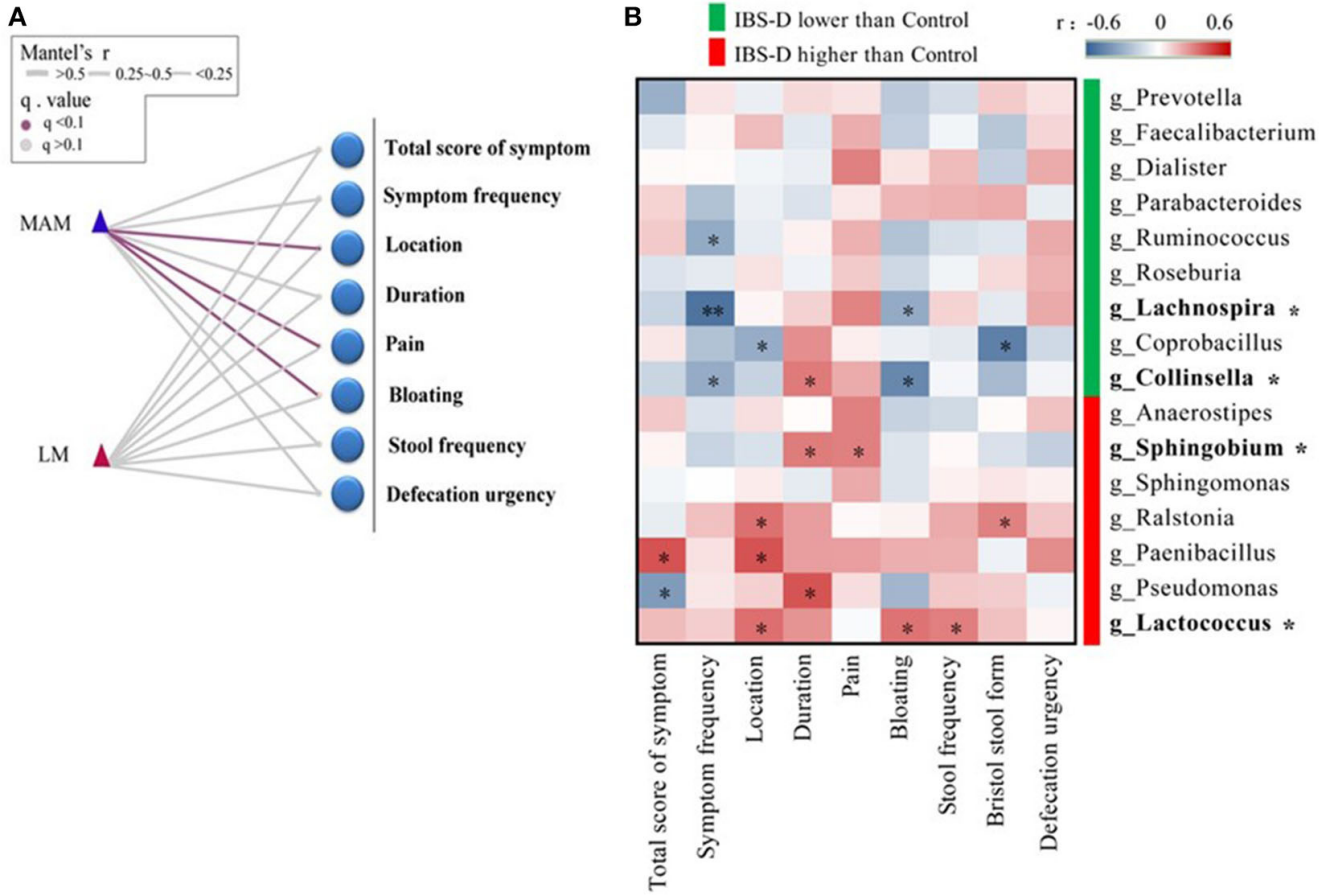
The correlation between the structure of microbiota and clinical manifestation in IBS-D patients. **(A)** Mantel's tests identified the correlation between phyla and clinical manifestation, which showed that MAM was significantly positively correlated with abdominal symptoms, abdominal pain, and bloating; however, LM was not associated with clinical manifestations. **(B)** The relationship between 16 predominant genera of MAM that differed significantly from HCs and clinical manifestation was analyzed by Spearman's correlation test, it was found that four predominant genera (with ^*^) were significantly correlated with abdominal pain and bloating. (location refers to left lower abdomen) (FDR was used to adjust the *P*-value).

We applied Mantel's tests to identify the correlation between microbiota (MAM and LM) at the phyla level and clinical manifestation of IBS-D (Figure 4A). The results showed that MAM was significantly positively correlated with abdominal symptoms (*r* = 0.21; *P* = 0.006), abdominal pain (*r* = 0.20; *P* = 0.019), and bloating (*r* = 0.13; *P* = 0.018); however, LM was not associated with clinical manifestations.

We used Spearman's correlation to explore the relationship between 16 predominant genera of MAM that differed significantly from HCs and clinical manifestation of IBS-D, it was found that four predominant genera were significantly correlated with abdominal pain and bloating (Figure 4B). *Lachnospira* and *Collinsella* were reduced in IBS-D patients and were negatively correlated with bloating (*Lachnospira*: *r* = −0.32; *P* = 0.043; *Collinsella*: *r* = −0.45; *P* = 0.013). *Sphingobium* and *Lactococcus* were increased in IBS-D patients; *Sphingobium* (*r* = 0.30; *P* = 0.047) was positively correlated with abdominal pain and *Lactococcus* (*r* = 0.33; *P* = 0.043) was positively correlated with bloating.

### Predicted Functional Genes in MAM and LM

**Figure 5:**
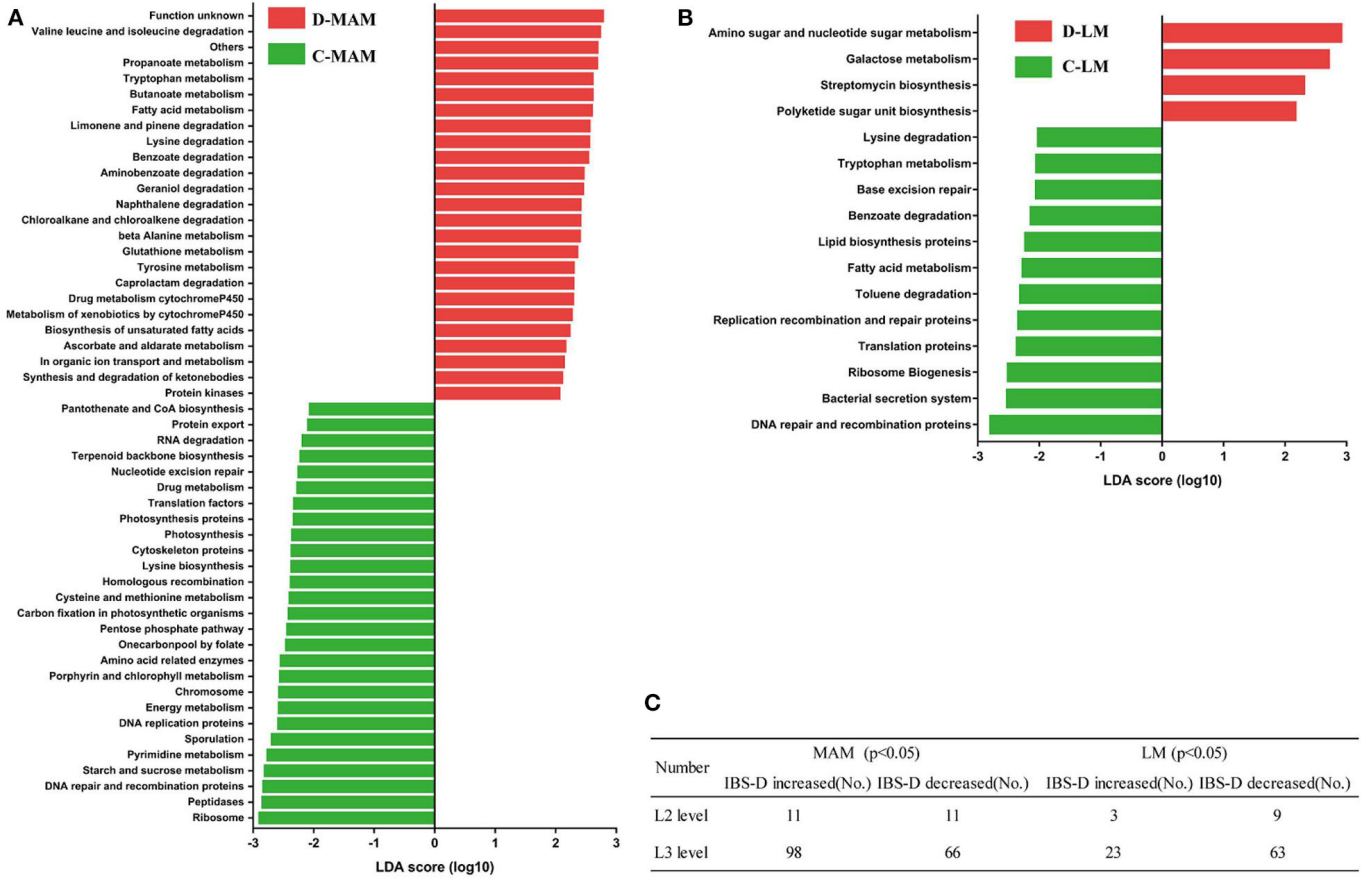
Functional genes with differences between IBS-D patients and HCs. (Red) taxa enriched in IBS-D patients, (Green) taxa enriched in HCs. The LDA scores of taxa over 2.5 are shown: **(A)** MAM and **(B)** LM. **(C)** The number of functional genes with a significant difference between IBS-D patients and HCs at L2 and L3 levels (C-LM, luminal microbiota in healthy controls; D-LM, luminal microbiota in IBS-D; C-MAM, mucosa-associated microbiota in healthy controls; D-MAM, mucosa-associated microbiota in IBS-D).

PICRUSt predicted 41 functional gene categories at the L2 level and 328 functional gene categories at the L3 level (Figure 5; Supplementary Table 2). MAM was found to be enriched in genes encoding enzymes mostly involved in amino acid metabolism (9.9%), whereas LM in membrane transport (11.3%) (Supplementary Table 2). In MAM, 22 functional genes altered in IBS-D and 12 in LM compared to HCs. The abundance of amino acid metabolism gene was decreased in MAM of IBS-D (10.2 vs. 9.9%; *P* = 0.24). A greater number of functional genes of MAM changed more than LM in IBS-D patients compared with HCs (L2 level: 22 vs. 12; L3 level: 164 vs. 86) and a similar trend was seen in the numbers of predominant functional genes (average abundance was over 0.1%) (15 vs. 7) (Figure 5; Supplementary Table 2).

### Correlation Between Functional Genes and Clinical Manifestation

**Figure 6:**
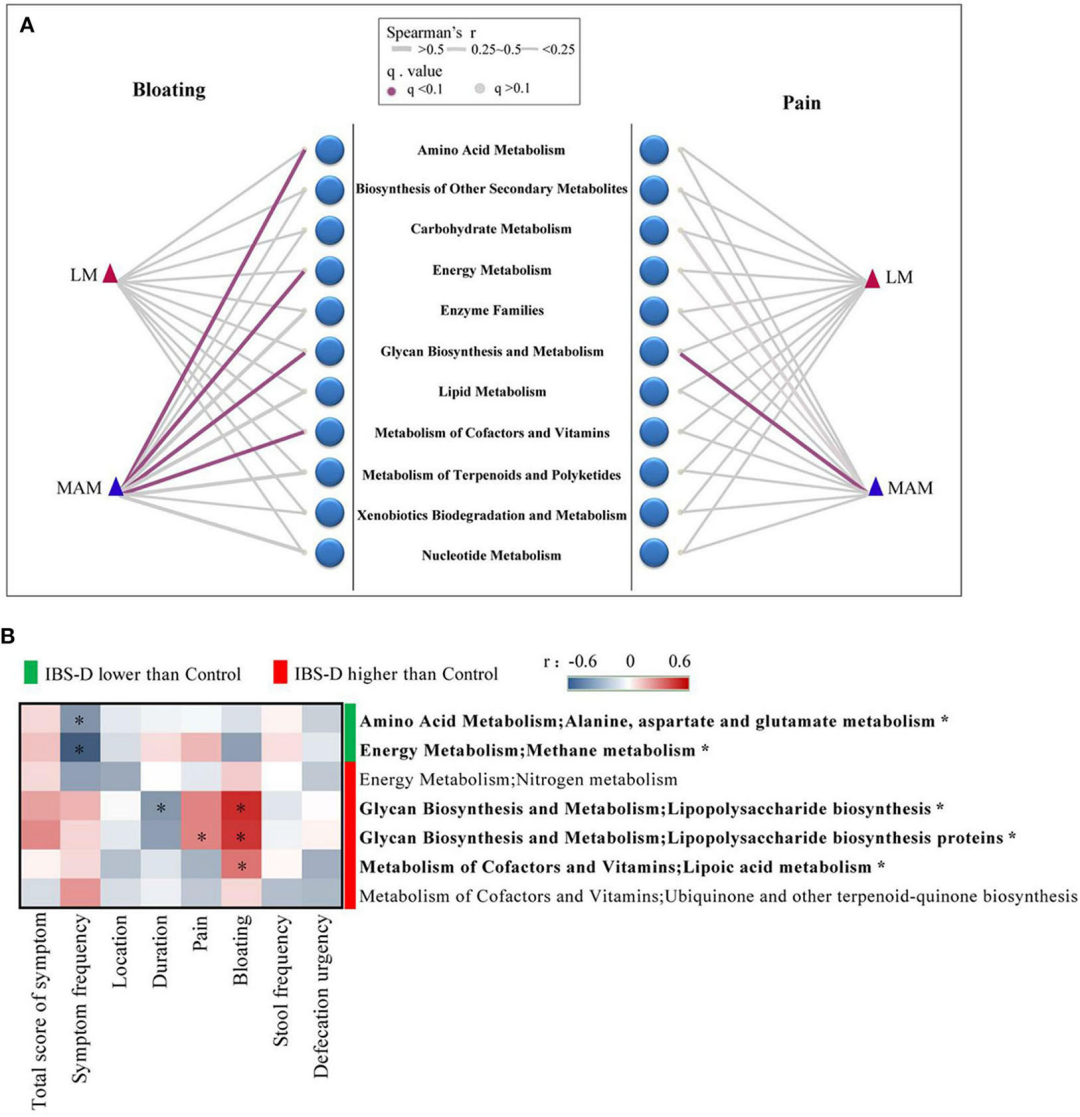
The correlation between metabolic pathways of functional genes and clinical manifestation. **(A)** The correlation between 11 metabolic pathways of functional genes and clinical manifestation, which showed four metabolic pathways in MAM were significantly correlated with abdominal pain and bloating. No metabolic pathway of functional genes in LM was significantly correlated with abdominal pain and bloating. **(B)** There were 7 metabolic pathways of functional genes belonged to the 4 metabolic pathways above showed a significant difference between IBS-D patients and HCs in MAM. The relationship between the 7 metabolic pathways and clinical manifestation revealed 5 metabolic pathways (with ^*^) had significant correlations with clinical symptoms of IBS-D disease in MAM (FDR was used to adjust the *P*-value).

We explored the relationship between 11 metabolic pathways of functional genes and abdominal pain and bloating. Four metabolic pathways of functional genes in MAM were significantly correlated with abdominal pain and bloating: abdominal bloating was negatively correlated with amino acid metabolism (*r* = −0.38; *P* = 0.033) and metabolism of cofactors and vitamins (*r* = −0.29; *P* = 0.042) and positively correlated with energy metabolism (*r* = 0.27; *P* = 0.04), glycan biosynthesis, and metabolism (*r* = 0.34; *P* = 0.035), and abdominal pain was positively correlated with glycan biosynthesis and metabolism (*r* = 0.26; *P* = 0.043). No metabolic pathway of functional genes in LM was significantly correlated with abdominal pain and bloating (Figure 6A).

We further found that seven functional genes belonged to the 4 metabolic pathways above showed a significant difference between IBS-D patients and HCs in MAM, while only the nitrogen metabolism in IBS-D patients was higher than HCs, the other metabolic pathway did not show any significant difference in LM (Supplementary Figure 1). Then we identified the relationship between the 7 metabolic pathways and clinical manifestation of IBS-D patients. In MAM symptom frequency was negatively correlated with alanine aspartate and glutamate metabolism (*r* = −0.32; *P* = 0.032) and methane metabolism (*r* = −0.48; *P* < 0.001), the duration of IBS-D was negatively correlated with lipopolysaccharide biosynthesis (*r* = −0.31; *P* = 0.038), abdominal pain was positively correlated with lipopolysaccharide biosynthesis proteins (*r* = 0.29; *P* = 0.049), bloating was positively correlated with lipopolysaccharide biosynthesis (*r* = 0.49; *P* < 0.001), lipopolysaccharide biosynthesis proteins (*r* = 0.47; *P* = 0.001), and lipoic acid metabolism (*r* = 0.33; *P* = 0.027) (Figure 6B). None of them in LM were correlated with bloating and abdominal pain (Supplementary Figure 2).

### Correlation Between Functional Genes and Predominant Microbiota

Four predominant genera and five functional genes were significantly correlated with the clinical symptoms of IBS-D in MAM. We analyzed the relationship between five functional genes and four predominant genera by functional annotations with the KEGG database, found *Collinsella* had functional genes encoding enzymes in alanine aspartate and glutamate metabolism, and methane metabolism, lipopolysaccharide biosynthesis, lipopolysaccharide biosynthesis proteins, and lipoic acid metabolism. *Lachnospira* had functional genes encoding enzymes in alanine aspartate and glutamate metabolism, methane metabolism, and lipopolysaccharide biosynthesis proteins. *Sphingobium* and *Lactococcus* expressed all five functional genes (Table 3).

**Table 3:** The expression of the 5 metabolic pathways genes in 4 predominant genera.

	**g_Lactococcus**	**g_Collinsella**	**g_Lachnospira**	**g_Sphingobium**
Alanine, aspartate and glutamate metabolism	**+**	**+**	**+**	**+**
Energy metabolism; methane metabolism	**+**	**+**	**+**	**+**
Lipopolysaccharide biosynthesis	**+**	**–**	**–**	**+**
Lipopolysaccharide biosynthesis proteins	**+**	**–**	**+**	**+**
Metabolism of cofactors and vitamins; lipoic acid metabolism	**+**	**–**	**–**	**+**

## Discussion

Our results showed significant differences between MAM and LM both in IBS-D patients and HCs; however, there were greater alterations of MAM than in LM in IBS-D patients compared to HCs. While the composition and predicted function genes of MAM were related to IBS-D clinical symptoms, the overall composition of LM did not change significantly and the structure and function of LM genes were not related to clinical symptoms. We also found that changes in intestinal MAM in IBS-D patients were closely related to abdominal pain and bloating. Four dominant genera in MAM were associated with abdominal pain and bloating. Two predicted function genes were positively correlated with abdominal pain and bloating and two other predicted function genes were negatively correlated with abdominal pain and bloating. We also found that five predicted function genes were closely related to the changes in four genera and that these four bacteria and related functional genes significantly changed in MAM but not in LM. Therefore, these results indicate that MAM plays a great role in the pathogenesis of IBS-D and should be highlighted in the future.

Consistent with previous studies (Carroll et al., 2012a; Ringel et al., 2015; Tap et al., 2017; Maharshak et al., 2018), we found significant differences between MAM and LM. Therefore, the relationship between MAM and LM and IBS-D clinical symptoms should be explained separately. There were greater changes in MAM compared to LM in IBS-D patients compared to HCs. MAM diversity was higher in IBS-D patients than LM and LM composition in IBS-D patients was not significantly altered, while MAM composition was significantly different compared to HCs. Two recent studies (Tap et al., 2017; Maharshak et al., 2018) indicated that changes in LM in IBS patients are unremarkable, consistent with our findings; these studies also found no differences in MAM between IBS patients and HCs. Tap et al. (2017) evaluated IBS-D, IBS with Mixed Bowel Habits (IBS-M), and IBS with Constipation (IBS-C) in MAM, which may have altered their findings, since each subtype may be associated with differences in MAM. Maharshak et al. (2018) studied IBS-D but only 14 samples were collected, limiting the sample size. A total of 46 mucosal samples were collected in our study, which represents the largest sample in a single IBS-D subtype study to date. Another research on IBS-C also found colonic mucosa other than fecal microbiota could discriminate patients with constipation from healthy individuals and fecal microbiota had no association with constipation (Parthasarathy et al., 2016). Similar results were reported in Inflammatory Bowel Disease (Altomare et al., 2019). We also found that a greater number of functional genes were changed in MMA compared to LM in IBS-D. MAM was more closer to intestinal epithelial cells and immune cells than LM, the microbiota and metabolic product of microbiota could be easier to effect on these cells directly. These findings indicated that MAM other than LM had a close relationship with IBS-D.

Our data indicated a close relationship between MAM and clinical symptoms but no correlation between LM and clinical symptoms in IBS-D. Our data showed that *Veillonella* and *Ruminococcus* in the fecal sample of IBS-D statistically changed compared to HC, which were consistent previous studies (Tana et al., 2010; Ringel et al., 2015; Chung et al., 2016), but we found there was no significant association with clinical symptoms. These studies recruited different subtypes of IBS that might amplify the influence of microbiota on IBS symptoms. Elevated fecal *Lactobacillus* and decreased *Bifidobacteria* reported to be related to abdominal symptoms or defecation (Ringel-Kulka et al., 2016; Zhong et al., 2019) were not found in this study which might be resulted from different methods of detection of the microbiota. Our results also showed that MAM was correlated with abdominal pain, bloating, and IBS-D duration. Specifically, the proportion of *Lachnospira* and *Collinsella* was decreased in IBS-D patients and was negatively correlated with bloating, while the proportion of *Lactococcus* and *Sphingobium* was increased in IBS-D patients and was positively correlated with bloating and abdominal pain, respectively. Decreased fecal *Lachnospiraceae* were reported to be associate with flatulence (Pozuelo et al., 2015; Ringel-Kulka et al., 2016). Studies have shown that intestinal *Lachnospira* belonged to the family of *Lachnospiraceae* and produced butyrate known to improve intestinal barrier function and inhibit intestinal inflammation (Ringel-Kulka et al., 2016; Simonyte Sjodin et al., 2016). Decreased mucosal *Lachnospira* might result in a shortage of butyrate on the surface of intestinal epithelial cells and dysfunction of intestinal barrier function. Kassinen et al. (2007) found that fecal *Collinsella* significantly decreased in IBS-D. *Collinsella* may affect intestinal carbohydrate uptake and intestinal gas production (Gomez-Arango et al., 2018). Previous data of *Lachnospira* and *Collinsella* were reported to decrease in LM of IBS, which differed with our data, however, these results suggest that *Lachnospira* and *Collinsella* may play a protective role in IBS-D, which were consistent with our data. Elevation of *Lactobacillus* in IBS was associate with abdominal symptoms for producing organic acid (Tana et al., 2010; Ringel-Kulka et al., 2016). Our data showed another organic acid-producing bacteria *Lactococcus* increased in IBS-D and a high concentration of organic acid levels associated with visceral hypersensitivity (Tana et al., 2010). *Sphingobium* was a Gram-negative bacterium (Takeuchi et al., 2001) which can produce lipopolysaccharides (LPSs), which increase intestinal sensitivity (Nozu et al., 2016); this increase in intestinal permeability and visceral sensitivity can further aggravate the symptoms of abdominal pain and bloating. We found increased proportions of *Lactococcus* and *Sphingobium* in MAM, which were positively correlated with bloating and abdominal pain; however, it remains unclear whether these bacteria play a role in the specific symptoms of IBS-D through these mechanisms. Therefore, we further evaluated alterations in MAM functional genes and their relationship with clinical symptoms.

We found that MAM function was closely related to clinical symptoms; however, there was no correlation between LM function genes and IBS-D clinical symptoms. The previous study reported alteration of fermentation, carbohydrate degradation, and amino acid pathways in the fecal microbiota of IBS (Ponnusamy et al., 2011; VichVila et al., 2018). Fecal metabolites such as tyrosine, glutamine conjugates (Jeffery et al., 2020), Short-chain fatty acids (Sun et al., 2019), methane (Tap et al., 2017), LPS were associated with IBS (Zhou S. Y. et al., 2018). Bloating was negatively correlated with amino acid and cofactor vitamin metabolism in MAM and positively correlated with energy metabolism and glycan biosynthesis and metabolism. Abdominal pain was positively correlated with glycan biosynthesis and metabolism. We also found that functional genes of glutamine and methane metabolism in MAM were decreased in IBS-D and were negatively correlated with the frequency of symptoms and bloating. These results suggested that glutamine metabolic function genes and methane metabolic genes play a protective role in IBS-D. The expression of LPS metabolic function genes and lipoic acid metabolic pathway genes in MAM was increased and was positively correlated with bloating and abdominal pain, suggesting that LPS metabolic function genes and lipoic acid metabolic pathway genes in MAM play a role in IBS-D. Glutamine supplementation can reduce the occurrence and clinical symptoms of post-infectious IBS (Zhou et al., 2018); additionally, methane has a protective effect on IBS-D patients (Pozuelo et al., 2015), which is consistent with our findings. Previous studies have found that methane metabolism genes are reduced in LM (Tap et al., 2017), while the results in MAM remain unclear. Through gene prediction function, we found that methane metabolism genes are reduced in MAM but not in LM, possibly due to different detection methods. A study of IBS-C found colonic mucosal but not fecal hydrogenogenic and hydrogenotrophic genes were more abundant in constipated patients (Wolf et al., 2017). The role of methane metabolism genes of MAM in IBS-D should be investigated further. Studies have also found that LPS can increase intestinal epithelial permeability and visceral hypersensitivity (Zhou S. Y. et al., 2018) and has a pro-inflammatory effect. LPS from the intestines can also affect the brain (Choi et al., 2012) through the blood-brain barrier, causing sensory abnormalities and further aggravating abdominal pain and bloating in IBS-D patients through the brain-gut axis. Lipoic acid is a promoter of some pathogenic bacteria (Zorzoli et al., 2016), which may increase the number of pathogenic bacteria. A study found a relative abundance of 262 virulence factors that contributed to the pathogenic potential of bacteria was increased in IBS compared with controls (VichVila et al., 2018). We found that the proportion of *Proteobacteria* (including most of the pathogenic bacteria) was significantly increased in the mucosa of IBS-D patients, and this increase in pathogenic bacteria may be related to an increase of lipoic acid synthesis, could worsen intestinal inflammation. These results suggest that an increase in LPS metabolic function genes and lipoic acid metabolism pathway genes in MAM might increase intestinal LPS and pathogenic bacteria and aggravate intestinal inflammation and visceral sensitivity. Therefore, glutamine metabolism, methane metabolism, LPS metabolism, and lipoic acid are involved in abdominal pain and bloating associated with IBS-D. The bacteria associated with these functions might represent a target for IBS-D therapeutics.

We further analyzed functional gene-related microflora by referring to the KEGG database and found that glutamine and methane metabolism was related to *Lachnospira* and *Collinsella*, LPS was related to *Lachnospira, Lactococcus*, and *Sphingobium*, and lipoic acid was related to *Lactococcus* and *Sphingobium*. Based on our data, *Lactococcus* was positively correlated with bloating and *Sphingobium* was positively correlated with abdominal pain. Therefore, we further speculate that *Lachnospira* and *Collinsella* play a protective role in IBS-D by affecting glutamine and methane metabolism, while *Lactococcus* and *Sphingobium* play a role in the clinical symptoms of IBS-D by affecting LPS and lipoic acid. These metabolic functional genes were changed in MAM but not in LM. Additionally, the bacteria associated with these functions, *Lachnospira, Collinsella, Lactococcus*, and *Sphingobium*, were significantly changed in MAM but not in LM of IBS-D patients, further indicating that MAM plays a more significant role in IBS-D compared to LM.

In this study, we evaluated the IBS-D subtype of IBS and harvested mucosal samples without bowel preparation to avoid its impact on MAM. We also used next-generation sequencing to explore the relationship between MAM and LM and the clinical manifestations of IBS-D from the two dimensions of flora structure and function. We found a close relationship between the structure and function of MAM and the clinical symptoms of IBS-D. A limitation of this study is that our data on metabolic function genes are derived from 16sRNA instead of the metagenome. Another limitation is not sex-matched between control and IBS-D patients. Additional research is required to evaluate LPS metabolism, methane metabolism, glutamine metabolism, and lipoic acid metabolism pathways of MAM, the mechanism underlying their involvement in IBS-D, and how to use these bacteria as therapeutic targets in the future.

## Data Availability Statement

The original contributions presented in the study are publicly available. This data can be found here: https://bigd.big.ac.cn/gsa-human/PRJCA002555.

## Ethics Statement

The studies involving human participants were reviewed and approved by Institutional Ethical Review committee of Huazhong University of Science and Technology. The patients/participants provided their written informed consent to participate in this study.

## Author Contributions

GL and XH: conceptualization, supervision, and writing—review and editing. YJ: data curation. MY and GH: formal analysis. XH: funding acquisition. MY: investigation and writing—original draft. YJ and YL: methodology. MY and YL: project administration. GH: software and visualization. All authors contributed to the article and approved the submitted version.

## Conflict of Interest

The authors declare that the research was conducted in the absence of any commercial or financial relationships that could be construed as a potential conflict of interest.
