# Supplementary material for: Mucosal-Associated Microbiota Other Than Luminal Microbiota Has a Close Relationship With Diarrhea-Predominant Irritable Bowel Syndrome
Source: Front Cell Infect Microbiol. 2020 Nov 2;10:515614. doi: 10.3389/fcimb.2020.515614 (PMC7667041; doi:10.3389/fcimb.2020.515614)
Supplement: Supplementary file 1 [file Table_1.DOCX]

**Supporting Online Materials**

**Figure S1**. The 7 metabolic pathways of functional genes in MAM and LM


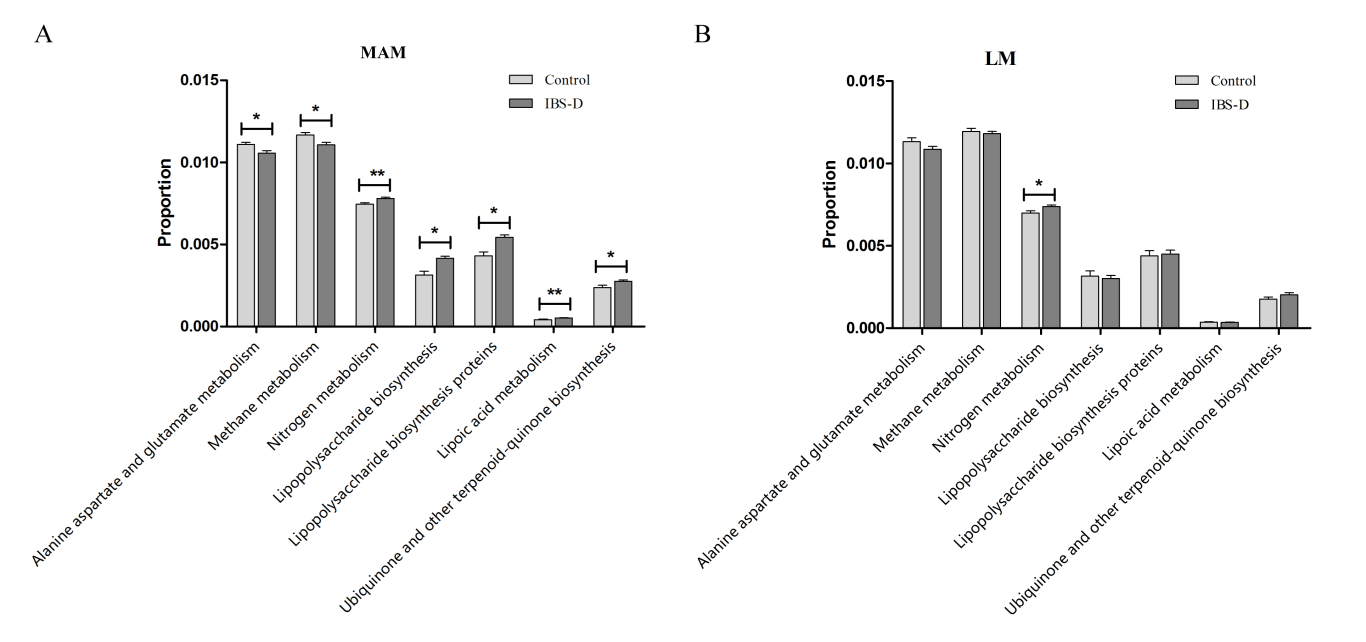


(A) In MAM there were 7 metabolic pathways of functional genes at the L3 level belonged to the 4 metabolic pathways above showed a significant difference between IBS-D patients and HCs, (B) while only the nitrogen metabolism in IBS-D patients was higher than HCs, the other metabolic pathway did not show any significant difference in LM.

**Figure S2.** The correlation between the 7 metabolic pathways of functional genes and clinical manifestation in LM


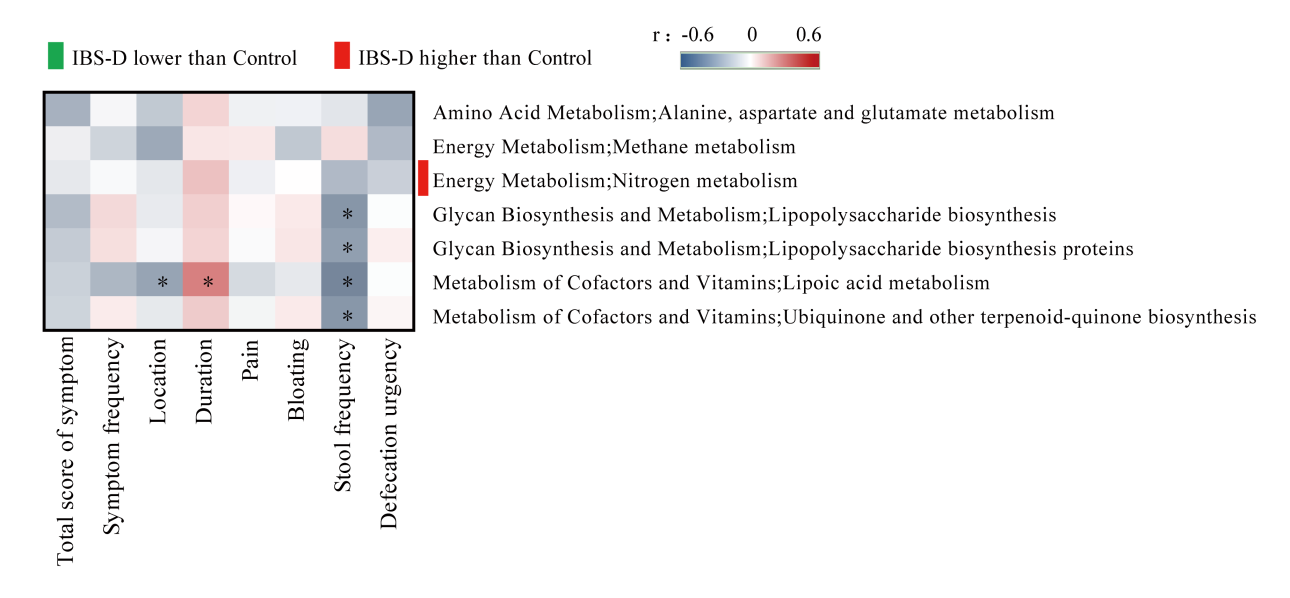


.

.

**Table S1** **Predominant genara with significantly difference between different groups**

| **MAM** | | | | |
| --- | --- | --- | --- | --- |
| genus | Control | IBS-D | p-value | q-value |
| *g__Dialister* | 0.01384 | 0.006006 | 0 | 0 |
| *g__Ruminococcus* | 0.010017 | 0.004232 | 0.001 | 0.008 |
| *g__Lactococcus* | 0.000814 | 0.012304 | 0.002 | 0.010667 |
| *g__Faecalibacterium* | 0.073633 | 0.039519 | 0.003 | 0.012 |
| *g__Sphingomonas* | 0.000805 | 0.004121 | 0.004 | 0.0128 |
| *g__Pseudomonas* | 0.000979 | 0.007282 | 0.013 | 0.034667 |
| *g__Lachnospira* | 0.004068 | 0.00192 | 0.017 | 0.034909 |
| *g__Collinsella* | 0.004063 | 0.003056 | 0.018 | 0.034909 |
| *g__Roseburia* | 0.009069 | 0.005265 | 0.02 | 0.034909 |
| *g__Paenibacillus* | 0.000327 | 0.006073 | 0.022 | 0.034909 |
| *g__Prevotella* | 0.169416 | 0.071683 | 0.024 | 0.034909 |
| *g__Ralstonia* | 0.000505 | 0.004606 | 0.028 | 0.037333 |
| *g__Anaerostipes* | 0.001317 | 0.00175 | 0.04 | 0.049 |
| *g__Sphingobium* | 0.0004 | 0.001568 | 0.047 | 0.049 |
| *g__Parabacteroides* | 0.017536 | 0.009864 | 0.048 | 0.049 |
| *g__Coprobacillus* | 0.002434 | 0.001016 | 0.049 | 0.049 |
| **LM** | | | | |
| genus | Control | IBS-D | p-value | q-value |
| *g__Haemophilus* | 0.000235 | 0.002974 | 0.006 | 0.036 |
| *g__Veillonella* | 0.00021 | 0.002026 | 0.022 | 0.048 |
| *g__Dorea* | 0.000511 | 0.003586 | 0.03 | 0.048 |
| *g__Sutterella* | 0.029358 | 0.022645 | 0.04 | 0.048 |
| *g__Dialister* | 0.046451 | 0.014386 | 0.044 | 0.048 |
| *g__Ruminococcus* | 0.028774 | 0.009018 | 0.048 | 0.048 |

**Table S2** **Predicting functions of microbiota with significantly difference between different groups at different levels**

| **MAM** | | | |  |
| --- | --- | --- | --- | --- |
| L2 | Control | IBS-D | p-value | q-value |
| Signal Transduction | 0.015441 | 0.017273 | 0.003 | 0.00825 |
| Folding, Sorting and Degradation | 0.025216 | 0.024131 | 0.018 | 0.0264 |
| Replication and Repair | 0.087259 | 0.078881 | 0 | 0 |
| Transcription | 0.026602 | 0.025347 | 0.012 | 0.020308 |
| Translation | 0.05437 | 0.047853 | 0 | 0 |
| Cardiovascular Diseases | 4.28E-05 | 8.45E-05 | 0.023 | 0.0275 |
| Metabolic Diseases | 0.001027 | 0.000941 | 0.005 | 0.012222 |
| Neurodegenerative Diseases | 0.001479 | 0.002046 | 0.021 | 0.027176 |
| Amino Acid Metabolism | 0.098761 | 0.101673 | 0.024 | 0.0275 |
| Energy Metabolism | 0.059292 | 0.057509 | 0.05 | 0.05 |
| Enzyme Families | 0.021707 | 0.020409 | 0.006 | 0.0132 |
| Lipid Metabolism | 0.030277 | 0.033881 | 0 | 0 |
| Metabolism of Other Amino Acids | 0.016418 | 0.017933 | 0.001 | 0.003667 |
| Metabolism of Terpenoids and Polyketides | 0.018262 | 0.019869 | 0.012 | 0.020308 |
| Nucleotide Metabolism | 0.040176 | 0.036926 | 0.001 | 0.003667 |
| Xenobiotics Biodegradation and Metabolism | 0.021178 | 0.029916 | 0.001 | 0.003667 |
| Circulatory System | 9.64E-05 | 0.000179 | 0.035 | 0.036667 |
| Environmental Adaptation | 0.001432 | 0.00131 | 0.002 | 0.006286 |
| Excretory System | 0.000265 | 0.000353 | 0.011 | 0.020308 |
| Immune System | 0.000871 | 0.000796 | 0.025 | 0.0275 |
| Genetic Information Processing | 0.025967 | 0.025124 | 0.02 | 0.027176 |
| Metabolism | 0.02498 | 0.026167 | 0.018 | 0.0264 |
|  |  |  |  |  |
| **LM** | | | |  |
| L2 | Control | IBS-D | p-value | q-value |
| Cell Communication | 8.55E-08 | 0 | 0.05 | 0.05 |
| Cell Growth and Death | 0.005206 | 0.0047 | 0.003 | 0.036 |
| Transport and Catabolism | 0.003549 | 0.003022 | 0.027 | 0.046286 |
| Membrane Transport | 0.112558 | 0.121357 | 0.033 | 0.0495 |
| Replication and Repair | 0.091167 | 0.085582 | 0.007 | 0.036 |
| Transcription | 0.027171 | 0.028663 | 0.016 | 0.036 |
| Translation | 0.057902 | 0.053608 | 0.018 | 0.036 |
| Metabolism of Terpenoids and Polyketides | 0.01681 | 0.01621 | 0.013 | 0.036 |
| Nucleotide Metabolism | 0.041661 | 0.039449 | 0.013 | 0.036 |
| Endocrine System | 0.003396 | 0.002997 | 0.041 | 0.05 |
| Sensory System | 2.85E-08 | 0 | 0.05 | 0.05 |
| Cellular Processes and Signaling | 0.042272 | 0.04459 | 0.048 | 0.05 |
|  |  |  |  |  |
| **MAM** | | | |  |
| L3 | Control | IBS-D | p-value | q-value |
| Butirosin and neomycin biosynthesis | 0.000675 | 0.000506 | 1.32E-05 | 0.000799 |
| Cell cycle - Caulobacter | 0.00507 | 0.004712 | 0.010704 | 0.016543 |
| Meiosis - yeast | 7.74E-05 | 0.000186 | 0.003789 | 0.008235 |
| p53 signaling pathway | 3.95E-05 | 8.00E-05 | 0.029064 | 0.031998 |
| Cytoskeleton proteins | 0.00351 | 0.003049 | 0.001196 | 0.004066 |
| Peroxisome | 0.002143 | 0.002411 | 0.019069 | 0.02446 |
| Phosphatidylinositol signaling system | 0.00091 | 0.00098 | 0.027588 | 0.031977 |
| Two-component system | 0.014056 | 0.015828 | 0.001445 | 0.004704 |
| Ion channels | 0.00013 | 0.00018 | 0.028259 | 0.031998 |
| Neuroactive ligand-receptor interaction | 4.75E-06 | 4.84E-08 | 0.044002 | 0.045183 |
| Proteasome | 0.000438 | 0.000393 | 0.015499 | 0.020801 |
| Protein export | 0.005893 | 0.005366 | 0.000269 | 0.001715 |
| RNA degradation | 0.004738 | 0.00445 | 0.014714 | 0.020281 |
| Sulfur relay system | 0.002445 | 0.002755 | 0.006131 | 0.011302 |
| Ubiquitin system | 0.000108 | 0.000162 | 0.003825 | 0.008235 |
| Base excision repair | 0.00433 | 0.004105 | 0.000118 | 0.00132 |
| Chromosome | 0.015687 | 0.014304 | 0.002574 | 0.007431 |
| DNA repair and recombination proteins | 0.027759 | 0.025382 | 9.21E-05 | 0.001281 |
| DNA replication | 0.006424 | 0.00574 | 0.000227 | 0.0016 |
| DNA replication proteins | 0.01212 | 0.010584 | 0.000153 | 0.001353 |
| Homologous recombination | 0.009141 | 0.008151 | 0.000351 | 0.001918 |
| Mismatch repair | 0.007832 | 0.006963 | 0.000166 | 0.001353 |
| Non-homologous end-joining | 0.000164 | 0.000266 | 0.023717 | 0.028349 |
| Nucleotide excision repair | 0.003803 | 0.003386 | 0.000294 | 0.00173 |
| RNA polymerase | 0.001501 | 0.001348 | 8.15E-05 | 0.001281 |
| Transcription machinery | 0.009956 | 0.009029 | 0.015139 | 0.020681 |
| Aminoacyl-tRNA biosynthesis | 0.011118 | 0.009898 | 1.96E-05 | 0.000799 |
| mRNA surveillance pathway | 2.56E-07 | 2.25E-06 | 0.012676 | 0.018325 |
| Ribosome | 0.022885 | 0.019803 | 2.07E-05 | 0.000799 |
| Ribosome Biogenesis | 0.013519 | 0.012177 | 6.43E-05 | 0.001281 |
| RNA transport | 0.001103 | 0.000969 | 0.020478 | 0.025497 |
| Translation factors | 0.005261 | 0.004547 | 3.32E-05 | 0.001016 |
| Bladder cancer | 2.77E-05 | 4.68E-05 | 0.018147 | 0.023731 |
| Colorectal cancer | 3.90E-05 | 7.93E-05 | 0.028865 | 0.031998 |
| Prostate cancer | 0.000416 | 0.000341 | 0.000667 | 0.002758 |
| Renal cell carcinoma | 8.89E-05 | 0.000133 | 0.014045 | 0.019535 |
| Small cell lung cancer | 3.90E-05 | 7.94E-05 | 0.02907 | 0.031998 |
| Viral myocarditis | 3.90E-05 | 7.94E-05 | 0.028774 | 0.031998 |
| African trypanosomiasis | 3.79E-05 | 8.63E-05 | 0.000536 | 0.002485 |
| Chagas disease (American trypanosomiasis) | 3.43E-05 | 7.42E-05 | 0.000741 | 0.002907 |
| Epithelial cell signaling in Helicobacter pylori infection | 0.000855 | 0.000714 | 0.000725 | 0.002907 |
| Pertussis | 0.000398 | 0.000537 | 0.044499 | 0.045389 |
| Staphylococcus aureus infection | 8.15E-05 | 0.000132 | 0.048556 | 0.048556 |
| Toxoplasmosis | 3.90E-05 | 7.93E-05 | 0.028825 | 0.031998 |
| Tuberculosis | 0.001548 | 0.00147 | 0.036421 | 0.038511 |
| Vibrio cholerae infection | 7.93E-07 | 2.15E-06 | 0.036497 | 0.038511 |
| Type I diabetes mellitus | 0.000562 | 0.000531 | 0.041816 | 0.043523 |
| Type II diabetes mellitus | 0.000465 | 0.00041 | 0.007843 | 0.013333 |
| Amyotrophic lateral sclerosis (ALS) | 0.000229 | 0.000351 | 0.012203 | 0.01811 |
| Huntington's disease | 0.00044 | 0.000647 | 0.008168 | 0.013687 |
| Prion diseases | 6.32E-05 | 9.21E-05 | 0.015321 | 0.020744 |
| Amino acid related enzymes | 0.014568 | 0.013383 | 7.83E-05 | 0.001281 |
| Cysteine and methionine metabolism | 0.009161 | 0.008567 | 0.001246 | 0.004144 |
| Lysine biosynthesis | 0.007718 | 0.006996 | 0.000259 | 0.001715 |
| Lysine degradation | 0.002094 | 0.003385 | 0.002655 | 0.007473 |
| Phenylalanine metabolism | 0.002413 | 0.003079 | 0.001094 | 0.003804 |
| Phenylalanine, tyrosine and tryptophan biosynthesis | 0.007797 | 0.007321 | 0.006591 | 0.011591 |
| Tryptophan metabolism | 0.002339 | 0.003863 | 0.004463 | 0.008845 |
| Tyrosine metabolism | 0.00369 | 0.004247 | 0.003485 | 0.008235 |
| Valine, leucine and isoleucine degradation | 0.003727 | 0.0059 | 0.003522 | 0.008235 |
| beta-Lactam resistance | 0.000289 | 0.000364 | 0.004357 | 0.008771 |
| Flavone and flavonol biosynthesis | 6.85E-05 | 4.44E-05 | 0.013218 | 0.018901 |
| Novobiocin biosynthesis | 0.001335 | 0.001284 | 0.019184 | 0.02446 |
| Penicillin and cephalosporin biosynthesis | 0.000347 | 0.000454 | 0.00912 | 0.014688 |
| Phenylpropanoid biosynthesis | 0.001642 | 0.001265 | 0.002915 | 0.00769 |
| Tropane, piperidine and pyridine alkaloid biosynthesis | 0.001248 | 0.001397 | 0.028846 | 0.031998 |
| Amino sugar and nucleotide sugar metabolism | 0.014421 | 0.013079 | 0.025959 | 0.030552 |
| Ascorbate and aldarate metabolism | 0.001205 | 0.001535 | 0.00105 | 0.0038 |
| Butanoate metabolism | 0.007107 | 0.00881 | 0.003167 | 0.008084 |
| Glyoxylate and dicarboxylate metabolism | 0.005872 | 0.006679 | 0.00019 | 0.001454 |
| Inositol phosphate metabolism | 0.001257 | 0.001549 | 0.003428 | 0.008235 |
| Propanoate metabolism | 0.005809 | 0.007603 | 0.00317 | 0.008084 |
| Pyruvate metabolism | 0.010292 | 0.010854 | 0.004903 | 0.009377 |
| Starch and sucrose metabolism | 0.009788 | 0.00826 | 0.001985 | 0.005955 |
| Carbon fixation in photosynthetic organisms | 0.006386 | 0.00578 | 0.003906 | 0.008235 |
| Methane metabolism | 0.011671 | 0.011072 | 0.024422 | 0.028966 |
| Nitrogen metabolism | 0.007452 | 0.007801 | 0.00632 | 0.011475 |
| Photosynthesis | 0.00391 | 0.003331 | 0.000136 | 0.00132 |
| Photosynthesis proteins | 0.004013 | 0.003468 | 0.000168 | 0.001353 |
| Sulfur metabolism | 0.002863 | 0.003112 | 7.54E-05 | 0.001281 |
| Cytochrome P450 | 1.69E-06 | 1.24E-05 | 0.020831 | 0.025497 |
| Peptidases | 0.018733 | 0.017086 | 0.002784 | 0.007473 |
| Protein kinases | 0.002972 | 0.003311 | 0.001744 | 0.005446 |
| Lipopolysaccharide biosynthesis | 0.003142 | 0.004159 | 0.019 | 0.02446 |
| Lipopolysaccharide biosynthesis proteins | 0.004319 | 0.005424 | 0.027 | 0.031534 |
| Peptidoglycan biosynthesis | 0.00805 | 0.007103 | 8.46E-05 | 0.001281 |
| alpha-Linolenic acid metabolism | 0.000112 | 0.000216 | 0.009586 | 0.01512 |
| Biosynthesis of unsaturated fatty acids | 0.0015 | 0.001989 | 0.011172 | 0.017051 |
| Fatty acid metabolism | 0.003472 | 0.005452 | 0.00366 | 0.008235 |
| Glycerophospholipid metabolism | 0.005236 | 0.004999 | 0.020767 | 0.025497 |
| Linoleic acid metabolism | 0.00067 | 0.000958 | 0.007772 | 0.013333 |
| Steroid hormone biosynthesis | 0.000278 | 0.000356 | 0.03106 | 0.033944 |
| Synthesis and degradation of ketone bodies | 0.000555 | 0.000986 | 0.008912 | 0.014538 |
| Lipoic acid metabolism | 0.000413 | 0.000527 | 0.005553 | 0.010489 |
| One carbon pool by folate | 0.006443 | 0.005717 | 0.000667 | 0.002758 |
| Pantothenate and CoA biosynthesis | 0.006065 | 0.005698 | 0.000294 | 0.00173 |
| Retinol metabolism | 0.00047 | 0.000699 | 0.000329 | 0.001864 |
| Thiamine metabolism | 0.004685 | 0.004301 | 0.002781 | 0.007473 |
| Ubiquinone and other terpenoid-quinone biosynthesis | 0.002377 | 0.002755 | 0.01721 | 0.022699 |
| Vitamin B6 metabolism | 0.002149 | 0.00201 | 0.016267 | 0.021642 |
| beta-Alanine metabolism | 0.002673 | 0.003796 | 0.00389 | 0.008235 |
| Cyanoamino acid metabolism | 0.003073 | 0.002761 | 0.013922 | 0.019535 |
| D-Alanine metabolism | 0.001026 | 0.000954 | 0.002721 | 0.007473 |
| D-Glutamine and D-glutamate metabolism | 0.001513 | 0.001434 | 0.033006 | 0.035563 |
| Glutathione metabolism | 0.00263 | 0.003319 | 0.000466 | 0.0023 |
| Taurine and hypotaurine metabolism | 0.001213 | 0.001318 | 0.003929 | 0.008235 |
| Biosynthesis of ansamycins | 0.000994 | 0.000875 | 0.012696 | 0.018325 |
| Biosynthesis of siderophore group nonribosomal peptides | 0.000331 | 0.000469 | 0.002199 | 0.00647 |
| Biosynthesis of type II polyketide backbone | 2.37E-05 | 2.55E-07 | 0.044946 | 0.045541 |
| Carotenoid biosynthesis | 0.000112 | 0.00017 | 0.03511 | 0.037565 |
| Geraniol degradation | 0.001284 | 0.002515 | 0.004251 | 0.008771 |
| Limonene and pinene degradation | 0.001617 | 0.002998 | 0.00645 | 0.011475 |
| Prenyltransferases | 0.003371 | 0.003067 | 0.003929 | 0.008235 |
| Sesquiterpenoid biosynthesis | 9.50E-06 | 6.35E-08 | 0.043319 | 0.044782 |
| Terpenoid backbone biosynthesis | 0.005772 | 0.005302 | 0.00023 | 0.0016 |
| Zeatin biosynthesis | 0.000581 | 0.000495 | 0.008932 | 0.014538 |
| Purine metabolism | 0.022099 | 0.020958 | 0.007342 | 0.012765 |
| Pyrimidine metabolism | 0.018077 | 0.015968 | 0.000138 | 0.00132 |
| 1,1,1-Trichloro-2,2-bis(4-chlorophenyl)ethane (DDT) degradation | 1.28E-05 | 2.95E-05 | 0.032836 | 0.035563 |
| Aminobenzoate degradation | 0.002043 | 0.003384 | 0.00363 | 0.008235 |
| Atrazine degradation | 0.000264 | 0.000458 | 0.011441 | 0.017162 |
| Benzoate degradation | 0.002925 | 0.00427 | 0.010203 | 0.015929 |
| Bisphenol degradation | 0.000958 | 0.001538 | 0.011256 | 0.017051 |
| Caprolactam degradation | 0.000738 | 0.001668 | 0.003743 | 0.008235 |
| Chloroalkane and chloroalkene degradation | 0.001814 | 0.002527 | 0.001565 | 0.004988 |
| Chlorocyclohexane and chlorobenzene degradation | 0.000333 | 0.000764 | 0.004567 | 0.008845 |
| Dioxin degradation | 0.000523 | 0.00073 | 0.019837 | 0.025083 |
| Drug metabolism - cytochrome P450 | 0.000678 | 0.001261 | 0.000599 | 0.002696 |
| Drug metabolism - other enzymes | 0.003322 | 0.002873 | 0.000121 | 0.00132 |
| Ethylbenzene degradation | 0.000585 | 0.000793 | 0.006444 | 0.011475 |
| Fluorobenzoate degradation | 0.000168 | 0.000404 | 0.009582 | 0.01512 |
| Metabolism of xenobiotics by cytochrome P450 | 0.000667 | 0.001188 | 0.001068 | 0.0038 |
| Naphthalene degradation | 0.001756 | 0.002556 | 0.004317 | 0.008771 |
| Styrene degradation | 0.000361 | 0.000633 | 0.005946 | 0.011094 |
| Toluene degradation | 0.001497 | 0.00184 | 0.00823 | 0.013687 |
| PPAR signaling pathway | 0.001444 | 0.001807 | 0.013558 | 0.019207 |
| Progesterone-mediated oocyte maturation | 0.000396 | 0.000327 | 0.000405 | 0.002066 |
| Renin-angiotensin system | 7.63E-06 | 2.23E-05 | 0.022536 | 0.02715 |
| Circadian rhythm - plant | 1.02E-05 | 2.50E-05 | 0.047332 | 0.047643 |
| Plant-pathogen interaction | 0.001422 | 0.001285 | 0.000618 | 0.002702 |
| Proximal tubule bicarbonate reclamation | 0.000264 | 0.000348 | 0.01231 | 0.01811 |
| Antigen processing and presentation | 0.000396 | 0.000327 | 0.000405 | 0.002066 |
| Hematopoietic cell lineage | 7.40E-07 | 7.51E-06 | 0.036852 | 0.038619 |
| RIG-I-like receptor signaling pathway | 3.48E-05 | 6.05E-05 | 0.00372 | 0.008235 |
| Inorganic ion transport and metabolism | 0.002282 | 0.002845 | 2.09E-05 | 0.000799 |
| Pores ion channels | 0.004542 | 0.005103 | 0.020776 | 0.025497 |
| Sporulation | 0.004734 | 0.003309 | 0.001785 | 0.005462 |
| Translation proteins | 0.008633 | 0.007862 | 0.000917 | 0.003422 |
| Biosynthesis and biodegradation of secondary metabolites | 0.000713 | 0.000822 | 0.022238 | 0.027003 |
| Metabolism of cofactors and vitamins | 0.00127 | 0.001508 | 0.000133 | 0.00132 |
| Others | 0.008941 | 0.009899 | 0.000806 | 0.003083 |
| Function unknown | 0.012869 | 0.014338 | 0.00053 | 0.002485 |
| General function prediction only | 0.037031 | 0.036081 | 0.004567 | 0.008845 |
|  |  |  |  |  |
| **LM** | | | |  |
| L3 | Control | IBS-D | p-value | q-value |
| Betalain biosynthesis | 8.37E-07 | 8.38E-08 | 8.26E-05 | 0.003802 |
| Adherens junction | 2.85E-08 | 0 | 0.049653 | 0.049653 |
| Focal adhesion | 2.85E-08 | 0 | 0.049653 | 0.049653 |
| Tight junction | 2.85E-08 | 0 | 0.049653 | 0.049653 |
| Apoptosis | 3.91E-05 | 1.13E-05 | 0.000471 | 0.003802 |
| Cell cycle - Caulobacter | 0.005113 | 0.004664 | 0.020754 | 0.041508 |
| p53 signaling pathway | 1.69E-05 | 7.77E-07 | 0.000664 | 0.003802 |
| Bacterial motility proteins | 0.005779 | 0.007972 | 0.032885 | 0.047715 |
| Flagellar assembly | 0.002249 | 0.003349 | 0.0364 | 0.049653 |
| Regulation of actin cytoskeleton | 2.85E-08 | 0 | 0.049653 | 0.049653 |
| Peroxisome | 0.001958 | 0.001681 | 0.010414 | 0.033659 |
| Phagosome | 2.85E-08 | 0 | 0.049653 | 0.049653 |
| MAPK signaling pathway - yeast | 0.000529 | 0.000444 | 0.029367 | 0.047715 |
| Two-component system | 0.013437 | 0.015505 | 0.039862 | 0.049653 |
| Ion channels | 9.53E-05 | 0.000166 | 0.011614 | 0.033659 |
| Protein export | 0.006136 | 0.005677 | 0.006113 | 0.023809 |
| RNA degradation | 0.004939 | 0.004633 | 0.029837 | 0.047715 |
| Sulfur relay system | 0.0024 | 0.002774 | 0.011434 | 0.033659 |
| Base excision repair | 0.004492 | 0.004219 | 0.004671 | 0.020333 |
| DNA repair and recombination proteins | 0.029031 | 0.027352 | 0.012736 | 0.034025 |
| DNA replication | 0.006769 | 0.006223 | 0.011826 | 0.033659 |
| DNA replication proteins | 0.01279 | 0.011922 | 0.030893 | 0.047715 |
| Homologous recombination | 0.009713 | 0.008974 | 0.013417 | 0.034025 |
| Mismatch repair | 0.008185 | 0.007689 | 0.048665 | 0.049653 |
| Nucleotide excision repair | 0.003939 | 0.003602 | 0.041164 | 0.049653 |
| RNA polymerase | 0.001605 | 0.001472 | 0.028288 | 0.047715 |
| Transcription factors | 0.01533 | 0.017488 | 0.023151 | 0.042863 |
| Aminoacyl-tRNA biosynthesis | 0.011894 | 0.01084 | 0.023169 | 0.042863 |
| mRNA surveillance pathway | 1.26E-07 | 2.08E-08 | 0.013794 | 0.034025 |
| Ribosome | 0.024323 | 0.022005 | 0.01495 | 0.035074 |
| Translation factors | 0.005604 | 0.00514 | 0.015167 | 0.035074 |
| Colorectal cancer | 1.69E-05 | 7.52E-07 | 0.00066 | 0.003802 |
| Small cell lung cancer | 1.69E-05 | 7.52E-07 | 0.00066 | 0.003802 |
| Arrhythmogenic right ventricular cardiomyopathy (ARVC) | 2.85E-08 | 0 | 0.049653 | 0.049653 |
| Dilated cardiomyopathy (DCM) | 2.85E-08 | 0 | 0.049653 | 0.049653 |
| Hypertrophic cardiomyopathy (HCM) | 6.04E-07 | 3.45E-08 | 0.0014 | 0.0074 |
| Viral myocarditis | 1.69E-05 | 7.52E-07 | 0.000665 | 0.003802 |
| Bacterial invasion of epithelial cells | 7.80E-06 | 4.32E-05 | 0.037186 | 0.049653 |
| Influenza A | 1.69E-05 | 7.59E-07 | 0.000668 | 0.003802 |
| Toxoplasmosis | 1.69E-05 | 7.52E-07 | 0.00066 | 0.003802 |
| Vibrio cholerae pathogenic cycle | 0.000719 | 0.000866 | 0.01109 | 0.033659 |
| Type I diabetes mellitus | 0.000578 | 0.000522 | 0.013152 | 0.034025 |
| Alzheimer's disease | 0.000534 | 0.000459 | 0.00017 | 0.003802 |
| Parkinson's disease | 4.45E-05 | 1.05E-05 | 0.006472 | 0.023946 |
| Amino acid related enzymes | 0.015186 | 0.014441 | 0.047557 | 0.049653 |
| Clavulanic acid biosynthesis | 8.33E-08 | 0 | 0.002313 | 0.010698 |
| Indole alkaloid biosynthesis | 6.69E-07 | 5.51E-08 | 0.000222 | 0.003802 |
| Isoflavonoid biosynthesis | 9.36E-08 | 0 | 0.001967 | 0.009704 |
| Sulfur metabolism | 0.002699 | 0.002927 | 0.031794 | 0.047715 |
| Cytochrome P450 | 8.47E-07 | 5.24E-08 | 0.000165 | 0.003802 |
| Protein kinases | 0.002806 | 0.003255 | 0.0304 | 0.047715 |
| Glycosylphosphatidylinositol(GPI)-anchor biosynthesis | 6.62E-08 | 0 | 0.049653 | 0.049653 |
| Lipid biosynthesis proteins | 0.006154 | 0.005743 | 0.005807 | 0.023809 |
| D-Glutamine and D-glutamate metabolism | 0.001608 | 0.001489 | 0.025298 | 0.04566 |
| Pyrimidine metabolism | 0.019085 | 0.017713 | 0.031152 | 0.047715 |
| Drug metabolism - other enzymes | 0.003548 | 0.003227 | 0.032362 | 0.047715 |
| Nitrotoluene degradation | 0.000666 | 0.000856 | 0.022738 | 0.042863 |
| Polycyclic aromatic hydrocarbon degradation | 0.001227 | 0.001044 | 0.017286 | 0.037622 |
| Xylene degradation | 0.00042 | 0.000543 | 0.047072 | 0.049653 |
| Cardiac muscle contraction | 2.76E-05 | 9.65E-06 | 0.029093 | 0.047715 |
| Bile secretion | 1.13E-06 | 3.14E-07 | 0.018764 | 0.03857 |
| Gastric acid secretion | 7.12E-09 | 0 | 0.049653 | 0.049653 |
| Pancreatic secretion | 7.12E-09 | 0 | 0.049653 | 0.049653 |
| Salivary secretion | 7.12E-09 | 0 | 0.049653 | 0.049653 |
| Adipocytokine signaling pathway | 0.000882 | 0.000683 | 0.017794 | 0.037622 |
| PPAR signaling pathway | 0.001277 | 0.001077 | 0.016146 | 0.036206 |
| Renin-angiotensin system | 1.82E-06 | 1.15E-07 | 0.000267 | 0.003802 |
| Aldosterone-regulated sodium reabsorption | 2.97E-07 | 2.63E-09 | 0.010651 | 0.033659 |
| Endocrine and other factor-regulated calcium reabsorption | 7.12E-09 | 0 | 0.049653 | 0.049653 |
| Hematopoietic cell lineage | 2.52E-07 | 3.33E-08 | 0.000349 | 0.003802 |
| Leukocyte transendothelial migration | 2.85E-08 | 0 | 0.049653 | 0.049653 |
| Phototransduction - fly | 2.85E-08 | 0 | 0.049653 | 0.049653 |
| Translation proteins | 0.009203 | 0.00884 | 0.043641 | 0.049653 |
| Carbohydrate metabolism | 0.001655 | 0.001895 | 0.041277 | 0.049653 |
